# *Paguristione uniuropodus*, a new genus and a new species of Pseudioninae infesting hermit crabs from China (Crustacea, Isopoda, Bopyridae)

**DOI:** 10.3897/zookeys.577.6295

**Published:** 2016-04-05

**Authors:** Jianmei An, Qiuping Zhao, John C. Markham

**Affiliations:** 1 School of Life Science, Shanxi Normal University, Linfen, 041004, P. R. China; 2 Arch Cape Marine Laboratory, Arch Cape, Oregon 97102-0133, U.S. A.

**Keywords:** Paguristes, East China Sea

## Abstract

*Paguristione uniuropodus* **gen. n.**, **sp. n.** infests *Paguristes* sp. in the East China Sea. *Paguristione* **gen. n.** differs from the closely related genera *Pseudione* and *Pagurion* by its females having indistinct lateral plates on the last two pleomeres and its male with a long tapering pleon of six pleomeres, lacking both pleopoda and uropoda.

## Introduction

Bopyrid isopods infesting hermit crabs belong to the subfamilies Pseudioninae (branchial parasites) and Athelginae (dorsoabdominal parasites). An, Markham and Yu (2010), An, Williams and Yu (2011) and An, Li and Markham (2013) have reported a total of eight bopyrid species infesting hermit crabs in the South China Sea. Markham (1992) recorded six species of bopyrids infesting hermit crabs in Hong Kong. Boyko (2004) reported one such species from Taiwan. In Chinese waters as a whole, An (2006) reported ten species of bopyrids infesting hermit crabs. Currently, throughout Asia, 36 species are recorded infesting 48 hermit crabs from Asia (Table 1). Worldwide, McDermott, Williams and Boyko (2010) catalog 83 species of bopyrids infesting hermit crabs, of which 41 species in ten genera are branchial parasites. As hosts worldwide, 11 species of *Paguristes* are known to bear bopyrids (Table 2); their parasites, all branchially infesting members of the subfamily Pseudioninae, are in the genera *Asymmetrione*, *Pseudione*, *Parapagurion* and now the new genus *Paguristione*.

**Table 1.** Bopyrid isopods infesting hermit crabs in Asian waters.

Bopyrids	Hosts	Localities	References
**Subfamily Pseudioninae**			
*Asymmetrione asymmetrica* (Shiino, 1933)	*Clibanarius bimaculatus* (De Haan, 1849)	Japan	Shiino 1933
	*Clibanarius merguiensis* de Man, 1888	Thailand	Markham 1985a; Brunenmeister 1980
*Asymmetrione sallyae* Williams & Schuerlein, 2005	*Diogenes avarus* Heller, 1865	Singapore	Williams and Schuerlein 2005
*Bopyrissa dawydoffi* (Codreanu & Codreanu, 1963)	*Clibanarius merguiensis* de Man, 1888	Vietnam	Codreanu and Codreanu 1963
*Bopyrissa liberorum* Markham, 1985	*Clibanarius merguiensis* de Man, 1888	Thailand	Markham 1985a
*Bopyrissa pyriforma* (Shiino, 1958)	*Clibanarius bimaculatus* (De Haan, 1849)	Hong Kong	Markham 1982
	*Diogenes edwardsii* (De Haan, 1849)	Japan	Shiino 1958
*Bopyrophryxus branchiabdominalis* Codreanu, 1965	*Oncopagurus monstrosus* (Alcock, 1894)	Indonesia	Bourdon and Boyko 2005
	*Paragiopagurus acutus* (de Saint Laurent, 1972)	Philippines	Bourdon and Boyko 2005
	unidentified pagurid	Indonesia	Bourdon and Boyko 2005
*Pagurion arrosor* An, Li & Markham, 2013	*Dardanus arrosor* (Herbst, 1796)	China	An, Li and Markham 2013
*Pagurion tuberculata* Shiino, 1933	*Dardanus scutellatus* (H. Milne Edwards, 1848)	Japan	Shiino 1933
	*Dardanus aspersus* (Berthold, 1846)	China	An, Li and Markham 2013
*Pagurocryptella holthuisi* Boyko & Williams, 2010	*Solitariopagurus tuerkayi* McLaughlin, 1997	Japan	Boyko and Williams 2010
*Parapagurion calcinicola* Shiino, 1933	*Calcinus elegans* (H.Milne Edwards, 1836)	Japan	Shiino 1933
	*Calcinus linapropodus* Morgan & Forest, 1991	Japan	Shiino 1933
	*Paguristes monoporus* Morgan, 1987	Indonesia	Haig and Ball 1988
	*Paguristes* sp.	Thailand	Markham 1985a
	Pagurus aff. hedleyi or *kulkarnii*	Hong Kong	Markham 1992
*Parapseudione lata* Shiino, 1958	*Pagurus middendorffii* Brandt, 1851	Japan	Shiino 1958
*Propseudione rhombicosoma* Shiino, 1933	*Calcinus laevimanus* (Randall, 1840)	Japan	Shiino 1933
	*Calcinus morgani* Rahayu & Forest, 1999	Japan	Shiino 1933
*Pseudione calcinii* Shiino, 1958	*Calcinus latens* (Randall, 1840)	Japan	Shiino 1958
*Pseudione clibanaricola* Shiino, 1933	*Clibanarius bimaculatus* (De Haan, 1849)	Japan	Shiino 1933
*Pseudione hyndmanni* (Bate & Westwood, 1868)	*Pagurus* sp.	Japan	Shiino 1936
*Pseudione intermedia* Nierstrasz & Brender à Brandis, 1932	Lophopagurus (Australeremus) triserratus (Ortmann, 1892)	Japan	Shiino 1936
	*Pagurus* sp. ?	Japan	Nierstrasz and Brender à Brandis 1932
*Pseudione kensleyi* Williams & Schuerlein, 2005	*Clibanarius infraspinatus* Hilgendorf, 1869	Singapore	Williams and Schuerlein 2005
*Pseudione nobili* Nierstrasz & Brender à Brandis, 1923	*Trizocheles spinosus spinosus* (Henderson, 1888)	Indonesia	Nierstrasz and Brender à Brandis 1923
*Pseudionella attenuata* Shiino, 1949	*Pagurus* sp.	Japan	Shiino 1949
*Pseudionella spiropaguri* An, Li & Markham, 2013	*Spiropagurus profundorum* Alcock, 1905	China	An, Li and Markham 2013
	*Spiropagurus spiriger* (De Haan, 1849)	China	An, Li and Markham 2013
*Parasymmetrione tuberculineata* An, Markham & Yu, 2010	*Clibanarius corallinus* (H. Milne-Edwards, 1848)	South China Sea	An, Markham and Yu 2010
*Asymmetrione globifera* An, Markham & Yu, 2010	*Dardanus hessii* (Miers, 1884)	Beibu Gulf	An, Markham and Yu 2010
	*Spiropagurus* sp.	South China Sea	An, Markham and Yu 2010
**Subfamily Athelginae**			
*Allathelges pakistanensis* Kazmi & Markham, 1999	*Paguristes perspicax* Nobili, 1906	Pakistan	Kazmi and Markham 1999
Athelges akanoshimensis var. tenuibranchiatus Shiino, 1936	Lophopagurus (Australeremus) triserratus (Ortmann, 1892)	Japan	Shiino 1936
*Athelges japonicus* Shiino, 1958	*Pagurus constans* (Stimpson, 1858)	Japan	Shiino 1958
	*Pagurus lanuginosus* De Haan, 1849	Japan	Shiino 1958
	*Pagurus middendorffii* Brandt, 1851	Japan	Shiino 1958
*Athelges* sp.	*Trizopagurus strigatus* (Herbst, 1804)	Indonesia	Haig and Ball 1988

**Table 2.** Known bopyrids infesting *Paguristes* species with localities and references.

Bopyrids	Host	Type locality	References
*Asymmetrione aequalis* Pardo, Boyko & Mantelatto, 2009	*Paguristes tomentosus* H. Milne Edwards, 1848	Peru	Pardo et al. 2009
*Asymmetrione desultor* Markham, 1975	*Pagurus tortugae* Schmitt, 1933	Brazil	Bourdon 1979
*Asymmetrione foresti* (Bourdon, 1968)	*Paguristes eremita* (Linnaeus, 1767)	Mediterranean	Bourdon 1968
*Parapagurion calcinicola* Shiino, 1933	*Paguristes monoporus* Morgan, 1987	Indonesia	Haig and Ball 1988
	*Pagurus* sp.	Thailand	Markham 1985a
*Parapagurion imbricata* Markham, 1978	*Pagurus tortugae* Schmitt, 1933	Cuba	Markham 1978
*Pseudione biacuta* Bourdon, 1979	*Paguristes robustus* Forest & de Saint Laurent, 1967	Uruguay	Bourdon 1979
*Pseudione quasimodo* Boyko & Williams, 2004	*Paguristes grayi* Benedict, 1901	Bahamas	Boyko and Williams 2004
	*Paguristes invisisacculus* McLaughlin & Provenzano, 1974	Bahamas	Boyko and Williams 2004
	*Paguristes anahuachis* Glassell, 1938	Gulf of California	Brusca 1980
*Allathelges pakistanensis* Kazmi & Markham, 1999	*Paguristes perspicax* Nobili, 1906	Pakistan	Kazmi and Markham 1999
*Athelges pelagosae* Babiç, 1912	*Paguristes eremita* (Linnaeus, 1767)	Adriatic	Babiç 1912
*Parathelges piriformis* Markham, 1972	*Paguristes oxyophthalmus* Holthuis, 1959	Colombia	Markham 1978
*Parathelges whiteleggei* Nierstrasz & Brender à Brandis, 1931	*Paguristes monoporus* Morgan, 1987	Indonesia	Haig and Ball 1988
*Pseudostegias otagoensis* Page, 1985	*Pagurus barbatus* (Heller, 1862)	New Zealand	Page 1985

Specimens used in this study were collected from the East China Sea in 1958, and one of the authors (An 2006) examined the parasites and reported *Parapagurion glabra* sp. n. infesting *Paguristes* sp. in her doctoral dissertation (not a published work in the sense of the ICZN). Further examination shows that they represent a new species in a new genus. The name *Parapagurion glabra* is here entered into its synonymy.

## Material and methods

Materials for this study originated from Chinese Comprehensive Oceanographic Survey. All materials examined have been deposited in the Institute of Oceanology, Chinese Academy of Sciences, Qingdao, China (IOCAS). Specimens were viewed and drawn using a Zeiss Stemi SV Apo microscope.

## Taxonomy

### Family BOPYRIDAE Rafinesque-Schmaltz, 1815 Subfamily Pseudioninae R. Codreanu, 1967

#### Paguristione gen. n.

Taxon classificationAnimaliaIsopodaBopyridae

http://zoobank.org/1076C4FA-D580-45AC-A918-B28C2D1FC396

##### Diagnosis.

Female. All body segments distinct, almost symmetry. Rudimentary coxal plates present in first four segments. Marsupium complete. Oostegite 1 with simple tubercules on internal ridge. Palp of maxilliped with long setae. All pleomeres distinct. First three pleomeres with lateral plates and biramous pleopoda. Fourth and fifth pleomeres with biramous pleopoda, but lateral plates without lateral plates. Sixth pleomere without lateral plates, uropoda uniramous. Male. All segments distinct. First and last pereomeres respectively much broader than adjacent head and first pleomere. Pereopods of first pair smaller than those of following 3 pairs. Pleon elongate, of 6 distinct pleomeres. No pleopoda or uropoda.

##### Etymology.

Combination of the genus name of its host, *Paguristes* and bopyrid genus name *Ione*. Gender feminine.

##### Type species.

*Paguristione uniuropodus* sp. n., herein designated.

#### Paguristione uniuropodus sp. n.

Taxon classificationAnimaliaIsopodaBopyridae

http://zoobank.org/E7EECB56-131C-4682-825B-1CBE50B12DCE

Fig. 1

Parapagurion grabla An, 2006 (unpublished thesis): 30–31, fig. 8 (invalid name).

##### Material examined.

Infesting *Paguristes* sp. Institute of Oceanology, Chinese Academy of Sciences, Qingdao, China (IOCAS). Chinese Comprehensive Oceanographic Survey, East China Sea, Station 4081, 28°00'N, 128°30'E, 74m, 5 April 1958, Yulin Liao, coll. Institute of Oceanology, Chinese Academy of Sciences, Qingdao, China (IOCAS). 1♀ holotype, CIEA408101; 1 ♂, allotype, CIEA408102.

##### Description of holotype female.

**Figure 1.**
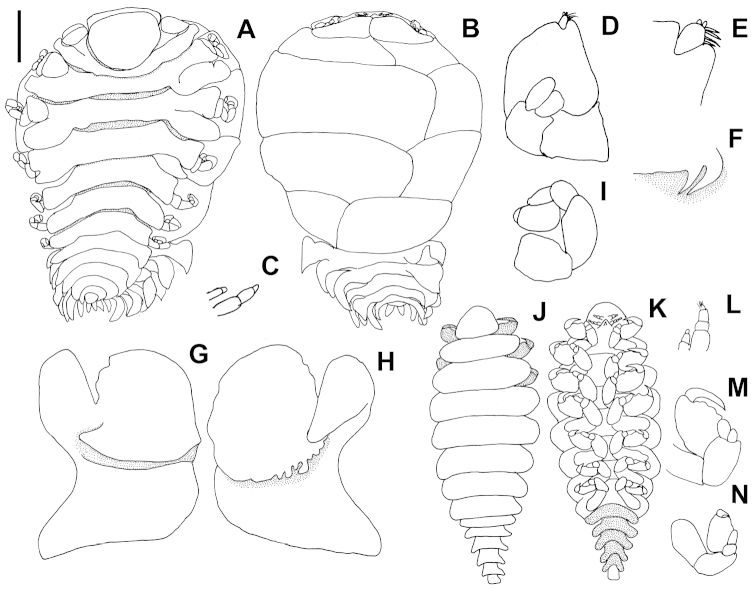
*Paguristione uniuropodus* sp. n. **A–I** holotype female **J–N** allotype male. **A** Dorsal view **B** Ventral view **C** Left antennae **D** Right maxilliped, external view **E** Palp of right maxilliped **F** Left side of barbula **G** Right oostegite 1, external view **H** Right oostegite 1, internal view **I** Pereopod 4 **J** Dorsal view **K** Ventral view **L** Left antennae **M** Pereopod 2 **N** Pereopod 7. Scale: 1.00 mm (**A, B**); 0.36 mm (**D**); 0.17 mm (**C, E**); 0.50 mm (**F–I**); 0.47 mm (**J, K**); 0.23 mm (L–M).

Length 5.20 mm, maximal width 3.41 mm across third pereomere, head length 1.0 mm, head width 1.31 mm. Body distorted about 16° (Fig. 1A).

Head subelliptical, fully embedded in pereomere 1, with short frontal lamina completely across anterior margin. Eyes absent (Fig. 1A). Antennae with two articles and three articles respectively (Fig. 1C). Maxilliped (Fig. 1D, E) with prominent round articulating palp, that fringed on medial margin by sparse setae. Plectron short and blunt. Barbula (Fig. 1F) with 2 large sharp falcate projections on each side, medially unornamented.

Pereon broadest across third pereomere. First 3 pereomeres with coxal plates. Brood pouch completely enclosed by oostegites. First oostegite (Fig. 1G, H) with deep groove separating 2 articles externally; internal ridge bearing 4-7 simple projections; posterolateral point extending laterally. Pereopods rudimentary, not extending beyond margins of brood pouch, visible only ventrally; all pereopods with all articles distinct, of nearly same size and structure (Fig. 1I).

Pleon of 6 distinct pleomeres, first three produced into small lateral plates and bearing biramous pleopods; fourth and fifth pleomeres lacking lateral plates. Terminal pleomere greatly reduced and deeply embedded in fifth, bearing uniramous uropoda. All pleopodal rami produced into tapering points and progressively smaller posteriorly, extending to sides of pleon and leaving ventral surface of pleon uncovered.

Description of allotype male

Body outline suboval. Length 2.52 mm, maximal width across third pereomere, 1.05 mm, head length 0.30 mm, head width 0.42 mm, first pleomere width 0.50 mm, fifth width 0.20 mm. All segments distinct (Fig. 1J, K).

Head semicircular, broader than long, much narrower than first pereomere, distinctly separated from first pereomere and not at all embedded into it (Fig. 1J). Eyes absent. Antennae visible only ventrally, not extending to margins of head, of 3 and 4 articles respectively; second antenna with sparse short setae on terminal article (Fig. 1L).

Pereon smoothly rounded, slightly broadest across third pereomere. No midventral tubercles. All pereopods with all articles distinct. Pereopod 1 somewhat smaller than pereopods 2-4, those 3 pairs largest and all of about same size; pereopods 5-7 progressively smaller (Fig. 1M, N). Pereopods 1-4 bearing sharp extended dactyli, dactyli of pereopods 5-7 much reduced.

Pleon elongate, extending far posteriorly, of 6 distinct pleomeres deeply separated laterally, each markedly narrower than that before it; pleomere 1 abruptly narrower than last pereomere, it and pleomere 2 much shorter than pleomeres 2-6; every pleomere broadest across posterior edge. Pleopods and uropods completely absent, not even indicated by scars.

##### Etymology.

Latin noun *uniuropodus*, referring to the uniramous uropoda of the female, used in apposition.

##### Remarks.

The new genus differs from other closely similar hermit-crab-infesting genera *Pseudione*, *Pagurion* and *Parapagurion* thus: female with only rudimentary pleonal lateral plates (only first three pleomeres with small lateral plates) and uniramous uropoda, male with head and pleon abruptly narrower than contiguous pereomeres, first pereopod smaller than pereopods 2-4 and pleopodal appendages completely lacking. Females of *Pseudione* have distinct pleonal lateral plates on pleomeres 1-5; its males have pleopods, and their heads and pleons are smoothly narrower than the pereon. Females of *Pagurion* have distinct lamellar pleopodal appendages on all pleomeres 1-6 and biramous uropoda; its males have equally width pereopods and uniramous pleopods. Females of *Parapagurion* are nearly symmetrical and bear well-developed lateral plates on pleomeres 1-5 and uniramous uropods; the first pereopods of the males are never smaller than the second ones.

## Supplementary Material

XML Treatment for Paguristione

XML Treatment for Paguristione uniuropodus
